# Optimized Unilateral Magnetic Resonance Sensor with Constant Gradient and Its Applications in Composite Insulators

**DOI:** 10.3390/s23125476

**Published:** 2023-06-09

**Authors:** Pan Guo, Chenjie Yang, Jiamin Wu, Zheng Xu

**Affiliations:** 1 College of Physics and Electronic Engineering, Chongqing Normal University, Chongqing 401331, China; 2019051107004@stu.cqnu.edu.cn; 2 Shenzhen Academy of Aerospace Technology, Shenzhen 518057, China; wujiamin@chinasaat.com; 3 School of Mechatronics Engineering, Harbin Institute of Technology, Harbin 150006, China; 4 School of Electrical Engineering, Chongqing University, Chongqing 400044, China; xuzheng@cqu.edu.cn

**Keywords:** aging assessment, composite insulator, transverse relaxation time (T_2_), unilateral magnetic resonance (UMR)

## Abstract

In this study, an optimized unilateral magnetic resonance sensor with a three-magnet array is presented for assessing the aging of composite insulators in power grids. The sensor’s optimization involved enhancing the static magnetic field strength and the homogeneity of the RF field while maintaining a constant gradient in the direction of the vertical sensor surface and maximizing homogeneity in the horizontal direction. The center layer of the target area was positioned 4 mm from the coil’s upper surface, resulting in a magnetic field strength of 139.74 mT at the center point of the area, with a gradient of 2.318 T/m and a corresponding hydrogen atomic nuclear magnetic resonance frequency of 5.95 MHz. The magnetic field uniformity over a 10 mm × 10 mm range on the plane was 0.75%. The sensor measured 120 mm × 130.5 mm × 76 mm and weighed 7.5 kg. Employing the optimized sensor, magnetic resonance assessment experiments were conducted on composite insulator samples utilizing the CPMG (Carr–Purcell–Meiboom–Gill) pulse sequence. The T_2_ distribution provided visualizations of the T_2_ decay in insulator samples with different degrees of aging.

## 1. Introduction

**Figure 1:**
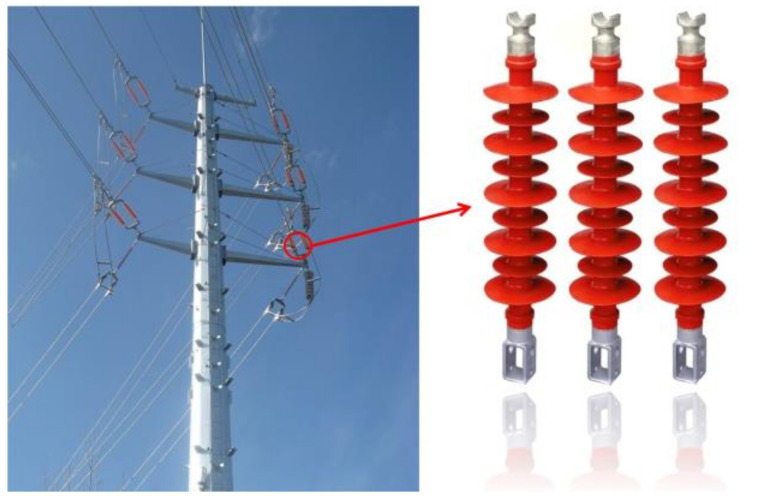
Composite insulators.

Insulators play a critical role in ensuring the safety of power systems [1]. As depicted in Figure 1, composite insulators have gained popularity in electric power systems because of their light weight, good anti-fouling and flash performance, and high mechanical strength. However, the prolonged exposure of composite insulators to electric fields, ultraviolet light, acid rain, and fouling leads to their aging and the deterioration of their electrical and mechanical properties, which potentially jeopardizes the power supply reliability of the power grid [2]. Therefore, testing the performance of composite insulators, assessing the degree of insulator aging, determining whether replacements are necessary, troubleshooting the power grid, and improving the efficiency of equipment condition maintenance are critical to ensuring the safe operation of the power system [3,4].

Unilateral magnetic resonance (UMR) sensors, comprising an open permanent magnet structure and surface radio frequency (RF) coils, offer a sensitive volume external to the sensor, facilitating the non-invasive investigation of objects of any size [5,6,7]. This feature enables a broad range of industrial applications, particularly when paired with a compact magnetic resonance (MR) console, creating a portable MR system.

Over the past three decades, several designs of unilateral magnets have been proposed to generate a static magnetic field B_0_ [8,9,10,11,12]. These designs can be broadly classified into two categories based on the distribution of the static magnetic field. The first category of magnets creates a saddle point of B_0_, where the derivatives of B_0_ are nulled around the saddle point. This design allows for a large excitation volume and reduces the diffusive attenuation of gradients caused by molecular motion. The second category of magnets generates a linear B0 distribution, creating a constant gradient perpendicular to the magnet surface. This type of magnet produces a well-defined sensitive volume, typically consisting of a thin layer or a set of layers. As a result, these magnets offer spatial information that can be utilized to investigate layered objects.

NMR-MOUSE (Nuclear Magnetic Resonance–Mobile Universal Surface Explorer) and its variations have been developed to establish a strong gradient (above 20 T/m) in the static magnetic field B_0_ [13,14,15]. However, this strong gradient can cause a relatively large change in the magnetic field strength within the sensitive layer due to temperature and the presence of ferromagnetic objects. Additionally, it results in a thin sensitive layer, which can be problematic for applications that involve diffusive attenuation or motion-induced decay. To address this issue, an optimized magnet geometry for NMR-MOUSE was proposed in a recent study, reducing the gradient from 20 T/m to 1.87 T/m [16]. Another magnet design was suggested, which utilizes a shaped pole piece to create a well-controlled gradient ranging from 0.3 to 2.5 T/m [17]. The same team also presented a three-magnet array unilateral magnet design that is easy and safe to install and compact in size. The gradient of this magnet was reduced to 0.63 T/m, but the strength of B_0_ was also decreased to 0.047 T [16,17]. The characteristics of these sensors described above are that there is a strong magnetic field gradient when the magnetic field intensity is high, or that the magnetic field intensity is correspondingly considerably reduced when the magnetic field gradient is reduced to a certain value.

**Figure 2:**
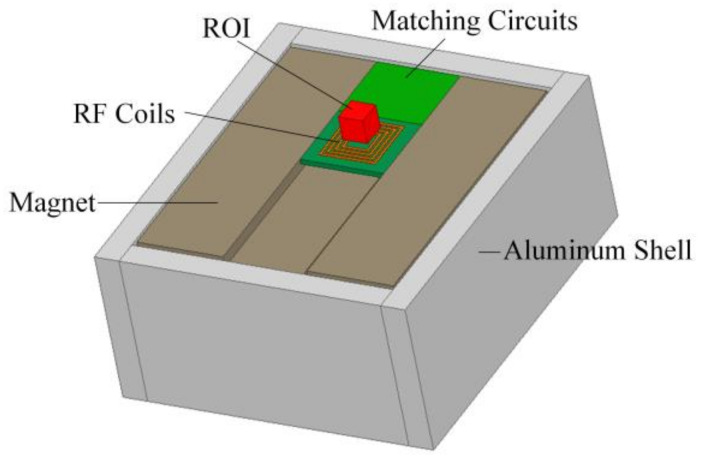
A schematic of the UMR sensor.

In this study, we aimed to optimize the design of a three-magnet array unilateral magnet to balance the strength of B_0_ and its constant gradient, achieving a larger sensitive volume while keeping a modest excitation bandwidth. This allows for the use of low-power RF amplifiers to enhance the mobility of the UMR system. The resulting UMR sensor (Figure 2) has dimensions of 120 mm × 130.5 mm × 76 mm and a mass of 7.5 kg. The center layer of the sensitive volume is positioned 4 mm away from the upper surface of the coil, and the magnetic field at the center point of the area is 0.139 T, with a gradient of 2.318 T/m. The corresponding hydrogen atomic nuclear magnetic resonance frequency is 5.95 MHz, and the uniformity of the magnetic field in the range of 10 mm × 10 mm on the plane is 0.75%.

The advantage of the sensor proposed in this paper is that it obtains a small magnetic field gradient while maintaining a certain magnetic field intensity and a small weight, so as to measure the sample with relatively small transverse relaxation time, as in the case of composite insulators. At the same time, in order to ensure the temperature stability of the static magnetic field of the sensor for engineering field measurement, a samarium cobalt magnet with high remanence temperature coefficient was employed. The sensor was compared with other similar sensors, as shown in Table 1. Additionally, the SNR of the sensor was improved by increasing the uniformity of the static magnetic field, increasing the uniformity and intensity of the RF magnetic field, and decreasing the eddy current effect. 

To validate the effectiveness of the optimized unilateral NMR sensor, we conducted assessment experiments on the aging of composite insulators using a prototype of the sensor. Insulator samples with varying levels of aging were set up, and NMR assessments were conducted by analyzing the relationship between the NMR signal and the sample’s aging time.

## 2. Materials and Methods

**Figure 3:**
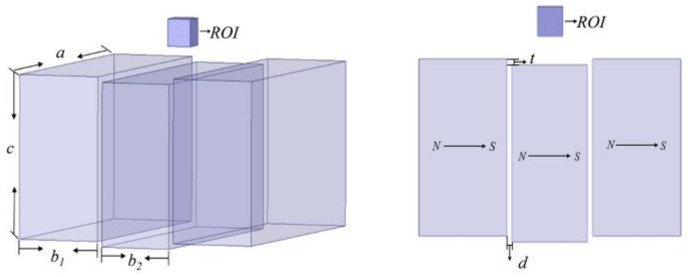
Schematic diagram of three-magnet array structure.

The sensor to be optimized is shown in Figure 3, which comprises of the magnet and the RF coil. According to Richards and Hoult [18,19], the signal-to-noise ratio (SNR) of NMR measurements can be expressed as:

(1) $$SNR=\frac{N\gamma^{3}\hbar^{2}I(I+1)}{6\sqrt{2}{\left(\kappa_{B}T\right)}^{\frac{3}{2}}}\cdot B_{0}^{2}\cdot \frac{V_{sample}}{\sqrt{\Delta f}}\cdot \frac{B_{1}/i}{\sqrt{R}}$$

To improve the SNR, under the condition that the tested sample and the experimental environment are determined, $V_{sample},B_{0}{}^{2},B_{1}/i$ should be maximized, and at the same time, $△f,\sqrt{R}$ minimized, where $B_{0}$ is the static magnetic field generated by the magnet and $B_{1}/i$ is the magnetic field generated by the RF coil when the unit current passes through. $V_{sample}$ is defined by the homogeneity of *B*_0_ and *B*_1_, which is usually called the ROI (region of interest), and $△f\;and\sqrt{R}$ are the bandwidth resistance of the RF coil. The magnet and RF coil optimization will be discussed as follows, respectively.

### 2.1. Magnet Optimization

Magnet optimization was carried out by calculating its magnetic field distribution. The remanent magnetization of a permanent magnet is equivalent to a toroidal current around the surface of the permanent magnet block, for which the magnetic field distribution of the permanent magnet can be calculated using the Biot–Savard law [20,21]. The magnetic field strengths *B_x_*, *B_y_*, and *B_z_* generated at any point P (x, y, z) in space can be expressed by Equations (2)–(6), where *dB_x_*, *dB_y_*, and *dB_z_* are the magnetic inductance components in the x, y, and z directions at point P.

(2) $$B_{x}=\int_{0}^{h}dB_{x}=-\frac{K}{2}\left[\begin{array}{l} \Gamma (a-x,y,z)+\Gamma (a-x,b-y,z)\\ -\Gamma (x,y,z)-\Gamma (x,b-y,z) \end{array}\right]{|}_{0}^{h}$$

(3) $$B_{y}=\int_{0}^{h}dB_{y}=-\frac{K}{2}\left[\begin{array}{l} \Gamma (b-y,x,z)+\Gamma (b-y,a-x,z)\\ -\Gamma (y,x,z)-\Gamma (y,a-x,z) \end{array}\right]{|}_{0}^{h}$$

(4) $$\begin{array}{l} B_{z}=\int_{0}^{h}dB_{z}=-K[\phi (y,a-x,z)+\phi (b-y,a-x,z)\\ +\phi (x,b-y,z)+\phi (a-x,b-y,z)+\phi (b-y,x,z)\\ +\phi (y,x,z)+\phi (a-x,y,z)+\phi (x,y,z)]{|}_{0}^{h} \end{array}$$

(5) $$\Gamma (\gamma_{1},\gamma_{2},\gamma_{3})=\ln\frac{\sqrt{\gamma_{1}{}^{2}+\gamma_{2}{}^{2}+{\left(\gamma_{3}-z_{0}\right)}^{2}}-\gamma_{2}}{\sqrt{\gamma_{1}{}^{2}+\gamma_{2}{}^{2}+{\left(\gamma_{3}-z_{0}\right)}^{2}}+\gamma_{2}}$$

(6) $$\varphi (\varphi_{1},\varphi_{2},\varphi_{3})=\left\{\begin{array}{l} \arctan\left[\frac{\varphi_{1}}{\varphi_{2}}\frac{\varphi_{3}-z_{0}}{\sqrt{\varphi_{1}{}^{2}+\varphi_{2}{}^{2}+{\left(\varphi_{3}-z_{0}\right)}^{2}}}\right]\\ 0 \end{array}\right.$$

where $K=\mu_{0}J/4\pi$ is the magnetic permeability in a vacuum and J is the surface current density. Γ is the notation of the functions of the independent variables $\gamma_{1}$,$\gamma_{2}$, and $\gamma_{3}$. φ is a functional of the independent variables *φ*1, *φ*2, and *φ*3. $[\cdot ]{|}_{0}^{h}$ denotes the subtraction of the function [·] between the values at *z* = h and *z* = 0. Therefore, the field strength at point P is:

(7) $$B=\sqrt{B_{x}{}^{2}+B_{y}{}^{2}+B_{z}{}^{2}}$$

**Figure 4:**
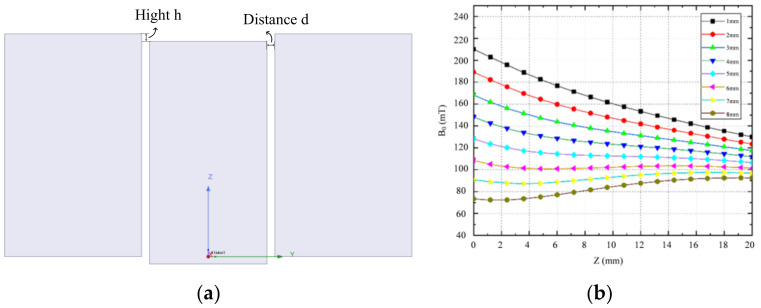
(**a**) Schematic diagram of the combined magnet, and (**b**) its magnetic field distribution along the *Z*-axis with h.

Figure 4a illustrates that the structure of the three-magnet combination is determined by the spacing d between the central magnet (with dimensions a = 120 mm, b = 30 mm, c = 60 mm) and the external magnet (with dimensions a = 120 mm, b = 35 mm, c = 60 mm), as well as the drop height h. For this study, the relative positions of the central and external magnets were adjusted to obtain a static magnetic field with a constant gradient in the target area. The spacing d was fixed at 2 mm, while the effect of the drop height h on the uniformity of the magnetic field in the target area was investigated. To simulate the magnet structure with different drop heights, the center of the upper surface of the magnet combination was used as the coordinate origin and located on the same plane as the upper surface of the external magnet. The magnetic induction intensity corresponding to different h values, distributed along the *Z*-axis, is shown in Figure 4b.

Based on the results shown in Figure 4b, it can be concluded that for drop heights of 1 mm, 2 mm, and 3 mm, the curve decreases linearly and maintains a constant magnetic field gradient within the target area. Additionally, to ensure a constant magnetic field gradient, it is important to also achieve a uniform magnetic field within the target plane (located at z = 13 mm). To simplify the process of optimizing the magnet, the magnetic field uniformity across the entire target plane is expressed as the uniformity of the two midlines (defined as xline and yline) along the X- and *Y*-axis directions.

(8) $$P=\frac{(B_{i}-B_{centre})\times 100}{B_{centre}}$$

where $B_{i}$ is the magnetic field strength at each point on the midline, and $B_{\mathrm{centre}}$ is the magnetic field strength at the center of the target surface.

**Figure 5:**
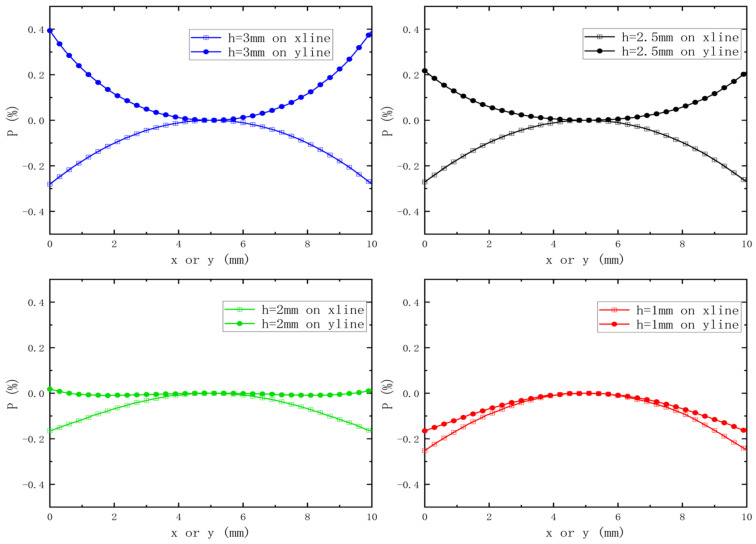
The variation law of the uniformity of the magnetic field of the two medians with a change in h.

The simulation results presented in Figure 5 show the changes in the magnetic field uniformity of the two central lines as a function of the drop height (h) of the three-magnet structure. As h decreases from 3 mm to 1 mm, the Yline uniformity changes from an upper concave to a lower concave shape, indicating the existence of an optimal value of h between 1 mm and 2 mm for achieving optimal magnetic field uniformity on the Yline. While the size of h also affects the magnetic field uniformity on the Xline, its effect is not significant. Based on several simulations, we determined that the optimal magnetic field uniformity on the target surface is achieved when h = 2 mm. Although the uniformity on the Xline is not as good as on the Yline, the effect is still significant compared to other h values.

**Figure 6:**
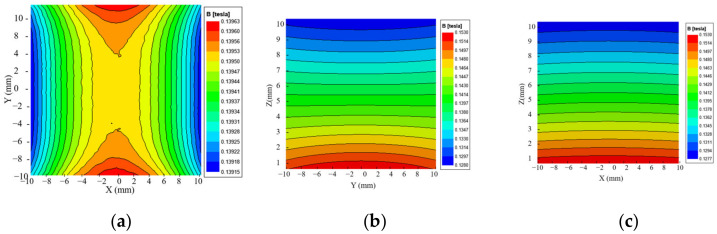
Magnetic field distribution in each plane at h = 2 mm. (**a**) XOY plane. (**b**) YOZ plane. (**c**) XOZ plane.

**Figure 7:**
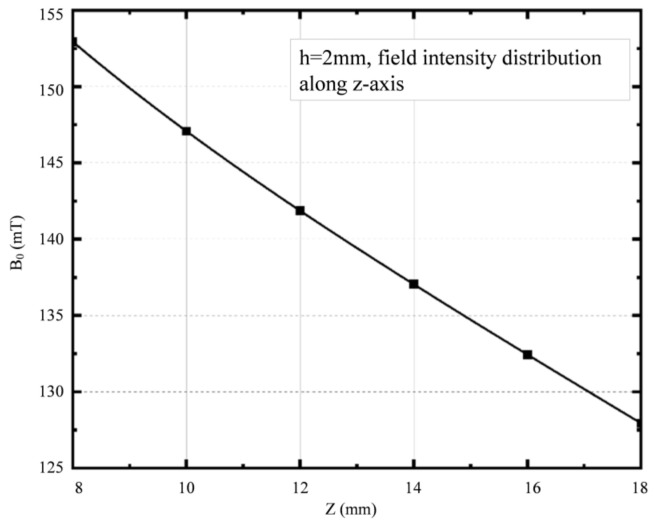
Magnetic field intensity distribution along *Z*-axis at h = 2 mm.

After analyzing the simulations of the magnet structure with different drop heights, a drop height of 2 mm was chosen between the central magnet and the external magnet to optimize the uniformity of the magnetic field. To verify the uniformity, the magnetic field distribution was simulated in each of the three planes of the target area. The magnetic field distribution at the center level is shown in Figure 6a, which reveals a magnetic field intensity of 139.74 mT at the center point with a deviation of 0.48 mT, resulting in a uniformity of 0.34% for this plane. Figure 6b,c present the magnetic field distribution at two perpendicular planes in the target area, showing that the contours are almost parallel, indicating that the magnetic field gradient along the *Z*-axis is a constant value. The magnetic field strength decreases from 153 mT to 128 mT in the target area, with a longitudinal gradient of 2.5 T/m, as shown in Figure 7.

### 2.2. RF Coil Optimization

When designing an RF coil to be used in conjunction with the current magnet, three practical considerations must be taken into account [22,23,24]. First, the coil must generate a sensitivity spot of 10 mm × 10 mm at z = 6 mm, where the magnet provides a homogeneous magnetic field on the XOY plane. Second, to prevent eddy current effects caused by close contact with the magnet, the wiring area of the RF coil must be restricted to 25 mm × 25 mm. Third, the coil inductance should be kept to a minimum to avoid possible detuning due to load changes introduced during the experiment. Therefore, the optimization objectives to obtain the optimal RF coil structure were chosen as B_1_ field intensity, excitation depth, and B_1_ uniformity on the XOY plane.

To optimize the RF coil, FEM simulation using commercial software such as Ansys Maxwell (ANSYS, Inc., Canonsburg, PA, USA) was employed. Based on the magnet optimization result, the nuclear magnetic resonance frequency is 5.95 MHz, which means that the wavelength of the electromagnetic wave emitted or received by the coil is 51.7 m. Due to the limited wiring area, the length of the coil winding is much less than the wavelength, and hence, the phase difference between the RF magnetic field generated by the RF coil passeing through AC and DC is negligible. To simplify the calculation, DC was used instead of AC to compute the B_1_ distribution in the simulation.

The simulation was carried out using the finite element method, with the current passing through the coil set to 1 A and the wire width and spacing both set to 1 mm. The number of turns in the coil was varied in the simulation, with values of 3, 4, 5, and 6 tested. The $B_{1}$-related parameters generated by different coil turns are shown in Table 2. The uniformity of the B_1_ field in the target XOY plane was defined as follows:

(9) $$U=2\frac{B_{1\max}-B_{1\min}}{B_{1\max}+B_{1\min}}\times 100\%$$

**Figure 8:**
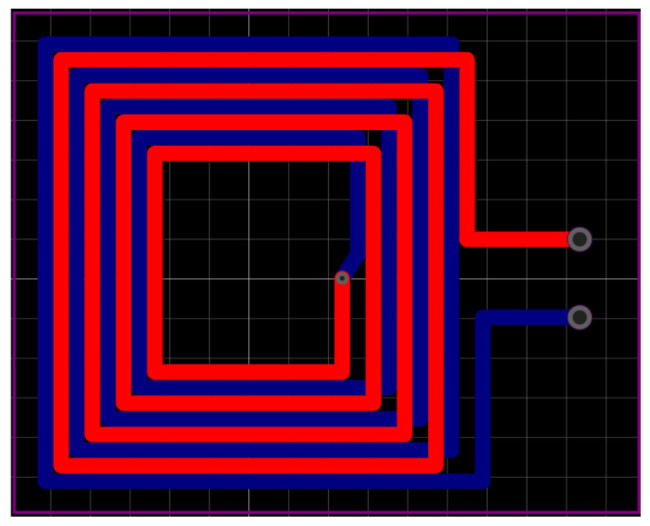
The two-layer coil wiring structure.

After considering the uniformity and strength of the *B*_1_ field, it was found that when the number of turns in the coil was set to 4, both the uniformity and strength were satisfactory. However, to generate a stronger magnetic field, the coil structure can be further modified. For instance, increasing the intensity of *B*_1_ can be achieved by transforming the single-layered coil into a double-layered coil, with each layer containing 4 turns. To minimize the distributed capacitance that may arise from the two parallel live wires, the wires of the second layer should be arranged alternately with the wires of the first layer, as depicted in Figure 8. 

The RF coils were fabricated on a PCB substrate, which is typically available in standard thicknesses of 0.2 mm, 0.4 mm, 0.8 mm, 1 mm, 1.6 mm, and 2 mm. To evaluate the effect of PCB thickness on the B_1_ field, simulations were conducted, and the results are presented in Table 3. As shown, the uniformity and intensity of the B_1_ field decrease with increasing PCB thickness.

**Figure 9:**
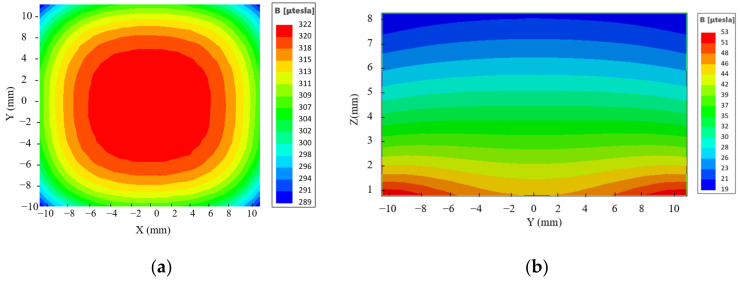
B_1_ distribution of the optimal RF coil. (**a**) XOY plane. (**b**) YOZ plane.

After considering various factors, such as B_1_ intensity and uniformity, excitation depth, and manufacturing difficulty, a double-layer coil with 4 turns and a 0.4 mm PCB thickness was chosen. The B_1_ field distribution for this coil configuration is shown in Figure 9. Finally, the optimal coil, which was made of copper with a thickness of 2 OZ, had two layers with four turns that were 1 mm wide. The distance between two leads was 1 mm, and that between the two layers was 0.4 mm.

### 2.3. Eddy Current Effect Optimization

**Figure 10:**
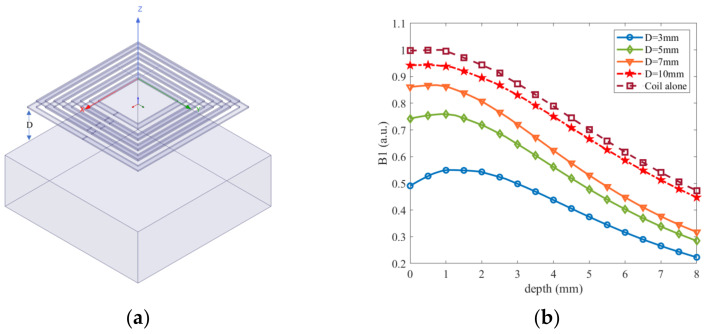
(**a**) Schematic diagram of the location of the RF coil and magnet, (**b**) Distribution curves of RF magnetic field along the *Z*-axis.

In order to optimize the coupling between the magnet and RF coil and minimize the impact of eddy currents on the *B*_1_ field strength, a 1 mm thick copper layer was used to shield the magnet. To investigate the effect of the RF coil and magnet on the *B*_1_ magnetic field and determine the different distances (D) between them, finite element simulations were conducted using a 30 mm × 30 mm × 10 mm copper cube instead of the magnet for simplicity. The D values chosen were 3, 5, 7, and 10 mm. Additionally, the *B*_1_ field when the RF coil operates independently was also included in the optimization process for comparison, to better evaluate the optimization results. Figure 10a presents a schematic diagram of the RF coil and magnet locations in the simulation.

The *B*_1_ profiles were measured for each D value, and the results are depicted in Figure 10b. The measurements revealed that the magnetic field intensity in the target region is highest when the RF coil is working alone, while the *B*_1_ field strength decreases with decreasing D values. At a depth of 4 mm, when D was set to 3 mm, only 24% of *B*_1_ was retained. However, when D was increased to 10 mm, *B*_1_ reached 95%, and the impact of the eddy current effect on the reduction in *B*_1_ became negligible. Thus, a value of D = 10 mm was selected as the final distance between the RF coil and the magnet.

## 3. Results and Discussion

### 3.1. Field Measurements

Fro magnets of the sizes given above, to improve the temperature stability of the magnetic field of the magnet, we employed (SmGd)_2_(CoFeCuZr)_17_ (YXG32, Ning-gang Permanent Magnetic Materials Co., Ltd., Ningbo, China) as the permanent magnet material since it has a low temperature coefficient (−0.035%/°C) and high remanence (Br 1.10–1.13 Tesla) between 20 °Cand 150 °C. The performance parameters of samarium cobalt permanent magnets are shown in Table 4.

**Figure 11:**
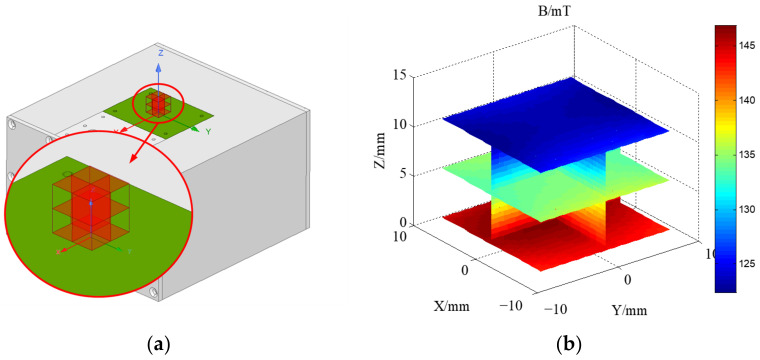
(**a**) Schematic diagram of the magnetic field test surface of the magnet. (**b**) Magnetic field distribution on the test surface.

**Figure 12:**
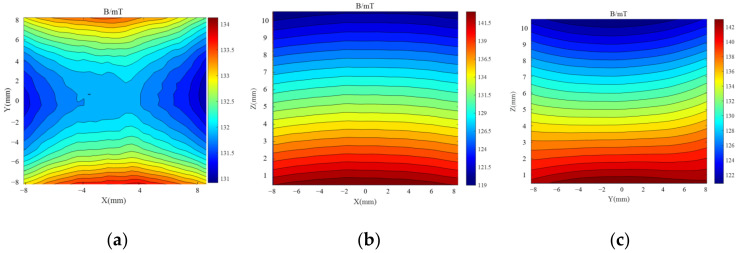
The B0 distribution. (**a**) Measuring plane z = 5 mm. (**b**) Measurement plane y = 0 mm. (**c**) Measuring plane x = 0 mm.

The BELL8030 Gauss meter (F.W. Bell Inc., Portland, OR, USA) and a computer-controlled 3-axis positioning system were utilized to measure the magnetic field of the optimized sensor prototype. The three-dimensional coordinates of the measurement points are depicted in Figure 11a, with the center of the upper surface of the RF coil serving as the origin of the coordinates, and the plane where the upper surface is located at z = 0 mm. A profile of the magnetic field distribution in the target area is illustrated in Figure 11b. Additionally, Figure 12 depicts the magnetic field distribution on three planes in the target area.

The center level of the target area was found to be 4 mm away from the upper surface of the coil, with a magnetic field at the center point of the area of 139.74 mT and a gradient of 2.318 T/m. This corresponds to the nuclear magnetic resonance frequency of hydrogen atoms of 5.95 MHz. Additionally, the magnetic field uniformity in the range of 10 mm × 10 mm on this level was calculated to be 0.75%.

### 3.2. Sensitivity Map

The area of the sensitive region of a unilateral NMR sensor is determined by the static magnetic field *B*_0_ and the RF field *B*_1_ with a complex functional relationship [25,26]. The calculation process will be briefly described below. The electric potential $\xi \left(t\right)$ induced in the coil can be written as:

(10) $$\xi \left(t\right)=\int \Phi \left(r\right)\left(\gamma B_{0}\left(r\right)\right)\left(\frac{\chi }{\mu_{0}}B_{0}\left(r\right)\right)\frac{B_{1}\left(r\right)}{I}F\left(\Delta \omega \left(r\right)\right)m_{xy}\left(r,t\right)dr$$

where Φ denotes the local spin density in the sample, γ is the spin ratio, χ is the nuclear magnetization, and $\chi =4.04\;\times \;10^{-9}$ in MKS; the first B0 term is the induction detection value based on Faraday’s law assumptions when measuring the NMR signal, and the second B0 term denotes the thermal longitudinal magnetization intensity $M_{0}\left(r\right)$; $B_{1}\left(r\right)/I$ denotes the magnetization efficiency of the receiver coil at point *r*; $F\left(\Delta \omega_{0}\left(r\right)\right)$ is the frequency response of the detection system, including the response of the tuned receiver coil and any hardware and or software filters; and $m_{xy}\left(r,t\right)$ denotes the transverse magnetization at point r and time t, normalized to $M_{0}\left(r\right)$. Thus, the main task of calculating the sensitivity region is to find $m_{xy}\left(r,t\right)$ a given pulse sequence in a static magnetic field.

Assuming that $\left|B_{0}\left(r\right)\right|\gg \left|B_{1}\left(r,t\right)\right|$ neglects the effects of relaxation and diffusion, the asymptotic CPMG signal can be described as in Equation (1).

(11) $$m_{axy}\left(\Delta \omega_{0},\omega_{1}\right)=\frac{\omega_{1}}{\Omega }\times \frac{\sin\left(\Omega t_{90}\right)}{1+{\left[\frac{\Omega }{\omega_{1}}\sin(\frac{\Delta \omega_{0}\cdot t_{E}}{2})\cot(\frac{\Omega \cdot t_{180}}{2})+\frac{\Delta \omega_{0}}{\omega_{1}}\cos(\frac{\Delta \omega_{0}\cdot t_{E}}{2})\right]}^{2}}$$

where $\Omega =\sqrt{\omega_{1}^{2}+\Delta \omega_{0}^{2}}$ is the chapter frequency of the spin during the RF pulse, and $\Delta \omega_{0}=\omega_{RF}-\gamma \left|B_{0}\right|$ and $\omega_{1}=\gamma \left|B_{1c}\right|$ are scalars, where *B*_1c_ is the component of the RF field *B*_1_ and this component is orthogonal to the main magnetic field *B*_0_.

(12) $$B_{1c}=\frac{1}{2}\left[B_{1}(r)-B_{0}(r)\frac{\left(B_{1}(r)\cdot B_{0}(r)\right)}{\left(B_{0}(r)\cdot B_{0}(r)\right)}\right]$$

**Figure 13:**
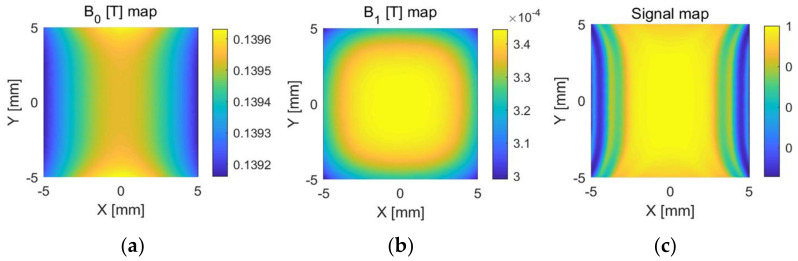
(**a**) Magnetic field distribution of B0. (**b**) Magnetic field distribution of B_1_. (**c**) Projection of the NMR sensitivity map onto the z = 4 mm plane.

To generate a sensitivity map for the unilateral NMR sensor, several steps were taken. First, vector maps of the main magnetic field *B*_0_ and the RF field *B*_1_ at the central level were obtained. Then, the value of *B*_1c_ was calculated for each point in the region using Equation (12). Next, the maximum value of *B*_1c_ (maxy) was calculated using Equation (11). Finally, maxy was substituted into Equation (10) to obtain the signal voltage, which was calculated using MATLAB software to generate the unilateral NMR sensitivity maps. The resulting maps are shown in Figure 13.

### 3.3. Aging Assessment of Composite Insulators

**Figure 14:**
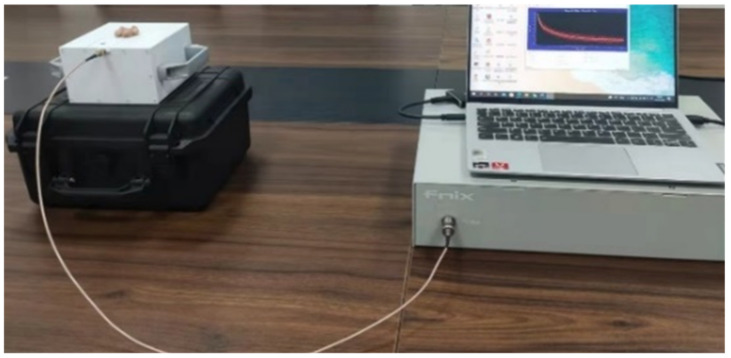
Assessment of aged insulator samples.

To evaluate the sensor’s capabilities, an experiment was conducted to assess the aging of composite insulators. The samples were aged inside a UV-accelerated aging chamber for 0 h, 24 h, and 48 h. These samples were placed in the central region of the RF coil of the unilateral NMR sensor, as depicted in Figure 14. The transverse relaxation time T2 of the aged insulators was measured using the Carr–Purcell–Meiboom–Gill (CPMG) sequence.

**Figure 15:**
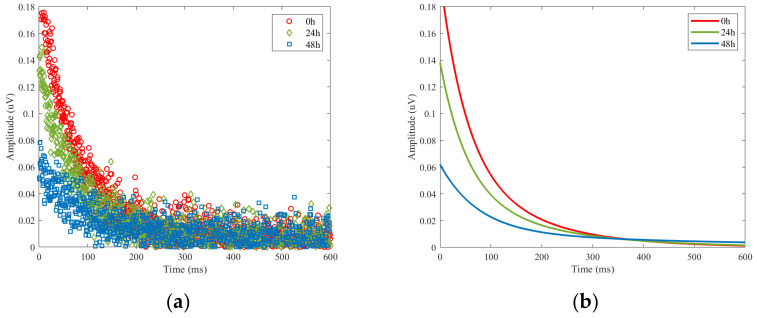
CPMG decay curve. (**a**) Data measured by the prototype. (**b**) The fitting curve of data in (**a**).

The experimental parameters were carefully selected and set. The resonant frequency used was f = 5.95 MHz, with the (π)⁄2 and π pulse attenuations set to −20 dB and −14 dB, respectively. Each point was sampled for 0.5 μs, with an echo time of 150 μs and a pulse length of 5 μs. The experiment utilized 32 sampling points and scanning was performed 30 times. The CPMG echo signal obtained from the experiment was fitted using a biexponential decay curve (Figure 15), from which two T2 values were obtained.

**Figure 16:**
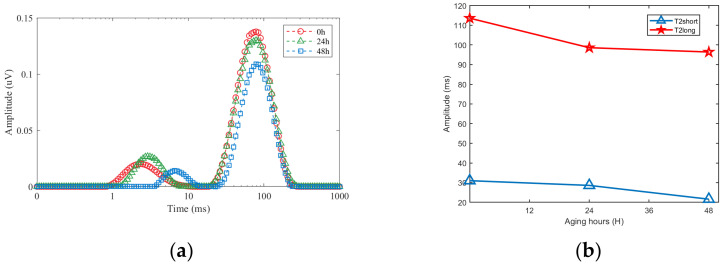
Analysis of NMR signal. (**a**) T_2_ distribution spectrum. (**b**) Insulator aging time and its corresponding T_2_.

Figure 16 shows the T_2_ trend obtained from assessments on insulators at different aging times. It can be seen that the amplitude of T_2_ decreases as the aging time of the insulator increases.

## 4. Conclusions

An optimized three-magnet array sensor is proposed for the aging assessment of composite insulators. The optimization process involves increasing the static magnetic field strength and the uniformity of the RF field, while also investigating and optimizing the effect of the eddy current between the RF coil and the magnet in the B_1_ field. The effectiveness of the optimized sensor is demonstrated through assessment experiments of aged insulators, where the transverse relaxation time T_2_ can be effectively analyzed for insulator samples with different aging levels using CPMG pulse sequences. The obtained transverse relaxation decay curves provide a fast and efficient method for assessing the aging levels of composite insulator samples.

## Figures and Tables

**Table 1:** Comparison of the sensor in this study with similar sensors.

Sensors	Frequency (MHz)	Gradient (T/m)	Dimension (cm)	Magnet Materials
NMR-MOUSE	17	20	20 × 20 × 10	Na_2_Fe_14_B
Mini-NMR-MOUSE	17.14	67.8	7 × 5 × 7	Na_2_Fe_14_B
Three-magnet array	4.26	2	10 × 8 × 5	N_48_NaFeB
The sensor in this work	5.95	2.318	12 × 13.05 × 7.6	(SmGd)_2_(CoFeCuZr)_17_

**Table 2:** $B_{1}$ related parameters generated by different coil turns.

Turns	$B_{1\max}\;\left(\mu T\right)$	$B_{1\min}\;\left(\mu T\right)$	*U*
3	111	109	99.05%
4	151	134	94.04%
5	184	144	87.80%
6	191	143	85.63%

**Table 3:** *B*_1_ related parameters generated by different thicknesses of PCB.

Thickness (mm)	$B_{1\max}\;\left(\mu T\right)$	$B_{1\min}\;\left(\mu T\right)$	U
0.2	322	289	94.50%
0.4	318	284	94.30%
0.8	313	278	94.08%
1.6	309	274	93.94%

**Table 4:** Performance parameters of samarium cobalt permanent magnets.

Model	Remanence	Coercivity	Intrinsic Coercivity	Maximum Magnetic Energy Product	Temperature Coefficient of Remanence
	$B_{r}\left(T\right)$	$H_{cb}\left(KA/m\right)$	$H_{cj}\left(KA/m\right)$	${\left(BH_{cb}\right)}_{max}\left(KA/m\right)$	$\left(\%/{}^{\circ}C\right)$
XYG-32	1.10–1.13	812–860	≥1433	230–255	−0.035

## Data Availability

The data that support the findings of this study are available from the corresponding author upon reasonable request.
